# The role of ageing in the wish to be dead: disentangling age, period and cohort effects in suicide ideation in European population

**DOI:** 10.1017/S2045796021000020

**Published:** 2021-02-18

**Authors:** M. Cabello, L. A. Rico-Uribe, J. C. Martinez-Ávila, A. Sánchez-Niubò, F. F. Caballero, G. Borges, B. Mellor-Marsá, J. M. Haro, M. Prina, S. Koskinen, J. L. Ayuso-Mateos

**Affiliations:** 1 Department of Psychiatry, Universidad Autonoma de Madrid, Madrid, Spain; 2 Instituto de Salud Carlos III, Centro de Investigación Biomédica en Red de Salud Mental (CIBERSAM), Madrid, Spain; 3 Instituto de Investigación Sanitaria Princesa (IIS-Princesa), Madrid, Spain; 4 Parc Sanitari Sant Joan de Déu, Fundació Sant Joan de Déu, Sant Boi de Llobregat, Barcelona, Spain; 5 Centro de Investigación Biomédica en Red de Epidemiología y Salud Pública, Madrid, Spain; 6 Department of Preventive Medicine, Public Health, and Microbiology, Universidad Autónoma de Madrid, Madrid, Spain; 7 National Institute of Psychiatry Ramon de la Fuente, Mexico City, Mexico; 8 Social Epidemiology Research Group. Health Service and Population Research Department, Institute of Psychiatry, Psychology & Neuroscience, King's College London, London, UK; 9 Global Health Institute, King's College London, London, UK; 10 National Institute for Health and Welfare (THL), Helsinki, Finland

**Keywords:** Cohort, Europe, period, suicide ideation

## Abstract

**Aims:**

To investigate potential age, period and birth cohort effects in the prevalence of suicide ideation in European ageing population.

**Methods:**

A total of 50 782 community-dwelling adults (aged + 50) from 20 different European countries were collected in the Survey Health Ageing and Retirement study. A multilevel logistic regression model of repeated measures was modelled to assess the effects of age and other variables, including the variability of observations over three levels: birth cohort groups, time period assessment and individual differences.

**Results:**

The larger effect of variability was attributed to individual-level factors (57.8%). Youngest-old people (65–79 years) showed lower suicide ideation than middle-aged people (50–64 years). No significative differences were found for suicide ideation between middle-aged people and oldest-old (80 + years). Only 0.85% and 0.13% of the total variability of suicide ideation accounted for birth cohort and period effects, respectively. Cohorts born between 1941 and 1944 possessed the lowest estimates of suicide ideation. Conversely, suicide ideation started to rise with post-War generations and reached a significant level for people born from 1953–1957 to 1961–1964. Regarding the time period, participants assessed in 2006–2007 showed a lower likelihood of suicide ideation. The rest of the cohorts and period groups did not show any significant effect on the prevalence of suicide ideation.

**Conclusions:**

Our results suggest that age and suicide ideation relationship is not linear in middle and older age. The European Baby boomers born from 50s to mid-60s might report higher suicide ideation than their ancestors. This scenario would imply a greater need for mental healthcare services for older people in the future.

## Introduction

The expression of suicide ideation has been longitudinally associated with completed suicide both in psychiatric and general population samples (Hubers *et al*., 2016). Suicide ideation is higher among people younger than 50 years in Europe (Bernal *et al*., 2007). However, people aged 50 years and older have a higher prevalence of completing suicide (Eurostat, 2020). In addition, suicide ideation in middle and old age is associated with functioning problems (Briggs *et al*., 2018), loneliness, poor social support (Almeida *et al*., 2012), and most importantly with an amount of psychological suffering that can be managed by existing treatments (O'Riley *et al*., 2014).

Although there are well-known risk factors for suicide ideation among middle and old age people, the role of ageing in this group is still unclear. Some studies have found that people younger than 65 years show higher suicide ideation than people older than 65 years (Cabello *et al*., 2019), whereas other studies have reported that suicide ideation increased with age among people older than 70 years (Stolz *et al*., 2016; Fässberg *et al*., 2020). These results might suggest a U-Shaped relationship between age and suicide ideation, increasing suicide ideation in the middle-aged, decreasing in the young-old and increasing again over the oldest-old. However, this available literature has several limitations that should be considered. First, the use of different measurements. Some measures include thoughts that life is not worth living (passive suicide ideation) (Stolz *et al*., 2016; Briggs *et al*., 2018), other instruments assess thoughts of taking own life (Cabello *et al*., 2019), and finally others mix both concepts (Almeida *et al*., 2012). Secondly, the inclusion of samples with different age groups. Some studies include age-heterogeneous samples (Briggs *et al*., 2018; Cabello *et al*., 2019), whereas others only include older people (Almeida *et al*., 2012; Fässberg *et al*., 2020). Finally, a third potential factor is probably related to the lack of distinction among age, cohort and period effects in most of the existing studies. Cohort effects refer to the existence of common cultural and societal values that are shared by people born in similar years. Period effects, in turn, are the environmental factors that are related to living in specific years and that affect all population age groups (for example, an economic recession or medical and technological advances). Cohort and period effects have been largely analysed for depression and suicide deaths (Phillips, 2014; Sullivan *et al*., 2020). However, only one study to our knowledge has identified that suicide ideation trends in the last two decades could be partially attributed to cohort and period effects (Twenge *et al*., 2019). In this study, people born after the 1980s reported higher suicide ideation rates than previous generations. In addition, an increasing suicide ideation trend was observed since 2015 across all age groups. Although this study is valuable, it did not include a large proportion of older adults, and it was limited to the US population.

Therefore, the present study is aimed at filling the existing gap in literature, by investigating longitudinally whether suicidal ideation trends in European ageing populations might respond to, age, time period, or birth cohort effects.

Besides clarifying previously mentioned inconsistent results, disentangling the role of age, period, and cohort effects on the prevalence of suicide would hold a number of different implications for suicide prevention. Firstly, given that approximately 35% of adults with suicide ideation continue to report suicide ideation 10 years later (Borges *et al*., 2008), the existence of a possible cohort birth effect among younger generations may help to predict a greater need for elderly mental health care as these generations age. On the other hand, an increasing prevalence of suicide ideation in the last few years due to a period effect might reveal that effective interventions should be launched to stem the increasing ‘epidemic’ of suicide ideation affecting all age groups. Finally, if the change is mainly due to the ageing process and the prevalence of suicide ideation lessens as the person ages, suicide ideation prevention efforts might be particularly indicated for specific lifetime periods.

## Methods

### Sample and study design

The Survey of Health, Ageing and Retirement in Europe (SHARE) is a cross-national panel survey including European community-dwelling population aged 50 and older (Börsch-Supan *et al*., 2013). A total of 50 782 participants from 20 European countries that participated at least once from Wave 1 to Wave 5 were included. Wave 1 was conducted in 2004–2005. Wave 2 was assessed during 2006–2007. Wave 4 information was mainly collected in 2011 and Wave 5 was carried out in 2013. Data from Wave 3 were excluded as they did not include any suicide ideation measure. Household response rates in baseline ranged from 40.3% (Belgium) to 97.5% (France) with an overall individual response rate of 60.1%. Household response rates by Wave and country can be consulted in supplementary material (online Supplementary Table S1). Wave-to-wave retention rates of Wave 1 participants was higher than 55% in all the countries (Bergmann *et al*., 2017). Data collection was conducted in the respondent's house using a computer-assisted personal interview. All interviewers were systematically trained by local group leaders who received a common train-the-trainer workshop. The study protocol was originally built in English and then translated using a forward-backward methodology. Special efforts were conducted to assure all the translations kept functional equivalence both in concepts and phrases used across countries. Further information on the study protocol development can be consulted elsewhere (Börsch-Supan *et al*., 2008). The study methods were approved by the University of Mannheim's internal review board until 2011 and by the Ethics Council of the Max-Planck-Society for the Advancement of Science since 2011. Further details of data collection, sampling methods and study design used are explained elsewhere for Wave 1 (Börsch-Supan *et al*., 2005), Wave 2 (Börsch-Supan *et al*., 2008), Wave 4 (Malter and Börsch-Supan, 2013) and Wave 5 (Malter and Börsch-Supan, 2015).

### Measures

#### Suicidal ideation

A binary item from the EURO-Depression scale (Prince *et al*., 1999) was used. The question included ‘In the last month, have you felt that you would rather be dead?’. Responses were coded as positive if any mention of wishing to be dead or suicidal feelings were reported and negative if there were no such feelings.

#### Health status

A common latent health score based on self-reported health items related to impairments in body functions, limitations in Activities of Daily Living and Instrumental Activities of Daily Living, and measured tests of cognitive performance and walking speed was created. This variable allowed us to control the effect that the general level of health might have on the presence of suicide ideation over time. Different statistical techniques (including Factor Analysis, Bayesian multilevel Item Response Theory and Machine Learning methods) were employed. The metric has shown a good performance in terms of predictive ability of mortality (de la Fuente *et al*., 2018) and morbidity (Caballero *et al*., 2017). Values ranged from 0 to 100, where higher values reflected a higher health status. Further information on this variable can be consulted elsewhere (Caballero *et al*., 2017).

#### Country

A total of 20 countries were included. Not all the countries participated in all the study assessments. There were some countries such as the Netherlands, Spain, Belgium, the Czech Republic, France, Germany, Italy, Sweden and Switzerland that participated in all the study waves, whereas others such as Hungary or Portugal only participated in Wave 4. The number of participants by wave in each country can be consulted in the supplementary material (online Supplementary Table S2).

#### Covariates

Age which was grouped into three categories (50–64, 65–79 and 80 and older), gender and level of education, divided into four categories (less than primary education, primary, secondary, and tertiary or higher education) and marital status grouped into four categories (single, married-cohabiting, separated-divorced, and widow) were also included.

### Analyses

Descriptive characteristics of the sample were described, using mean and standard deviation for continuous variables, and frequencies and percentages for categorical variables. Chi-squared tests were used in order to assess the association between categorical variables and the presence of suicidal ideation in Wave 1. Similarly, the Wilcoxon test was used for estimating differences between suicide ideation groups in continuous variables.

The measurement occasions were compilated as period effects including the following four groups of years: 2004–2005, 2006–2007, 2011 and 2013. Over this time period, a total of 15 consecutive birth cohorts were made every 4 years, beginning with those born in 1905–1908 and ending with those born in 1961–1964. We selected cohorts based on 4 years period to ensure a homogeneous and sufficient number in all groups. Prevalence rates of suicide ideation were calculated by birth cohort and period groups and graphically presented.

Next, to better disentangle the effects of age, time period, and birth cohort, we performed a mixed-effect model. Suicide ideation was recorded in SHARE as a dichotomous variable, for this reason a logistic approach was applied. The proposed model was a logistic mixed model of repeated measures which could be described as follows:
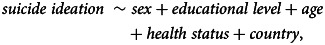
with 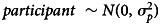, 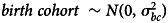 and 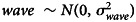 as random effects

Therefore, the random part of the model included three levels: the person, the birth cohort and the time period. Random effects at a personal level were added in order to capture variability due to intrinsic participant characteristics. Cohort birth was added as a random effect to explain the existence of cultural and societal changes that make people born in specific years more vulnerable to suicide ideation. Finally, the period effect was also included as random to account for variability due to the specific circumstances that all participants experienced over the study time. The identification problem, which is the linear dependence between age, period and cohort effects was managed according to Reither's indications (2015). We decided to include periods and cohorts as random since they are contextual variables, that are independent of individual-level variables. These random effects followed a normal distribution with a mean of zero and variance to be estimated, the larger the variance, the higher the impact in the observed variability. The fixed part of the model included sex, educational level, age, health status and country as fixed effects. Age was treated as fixed because it was considered as a biological factor that is related to individual-level characteristics and whose effects would be constant across individuals.

After running the Restricted Maximum Likelihood Model, estimated coefficients for fixed effects were transformed into odds ratios (ORs), with variance components estimations providing an idea of influence in variability since total variance can be described as follows, 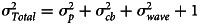.

Finally, considering the results of the previous modelled relationships, we simulated the posterior distributions of birth and periods effects on suicide ideation. Predicted OR and confidence intervals (95%) were generated from 500 simulated distributions, estimating the values of suicide ideation for each cohort and period level. Results were graphically presented.

All statistical analyses were done using R (R Core Team, 2013). All graphs were created using ggplot2 package (Wickham, 2016). Mixed-effect model was fitted using the glmer function in the lme4 package (Bates *et al*., 2015). Simulations of random effects were calculated using the REsim function in the merTools package (Knowles and Frederick, 2016). P values lower than 0.05 were considered significant. All the analyses were conducted with the available information with no data imputation.

## Results

**Fig. 1.**
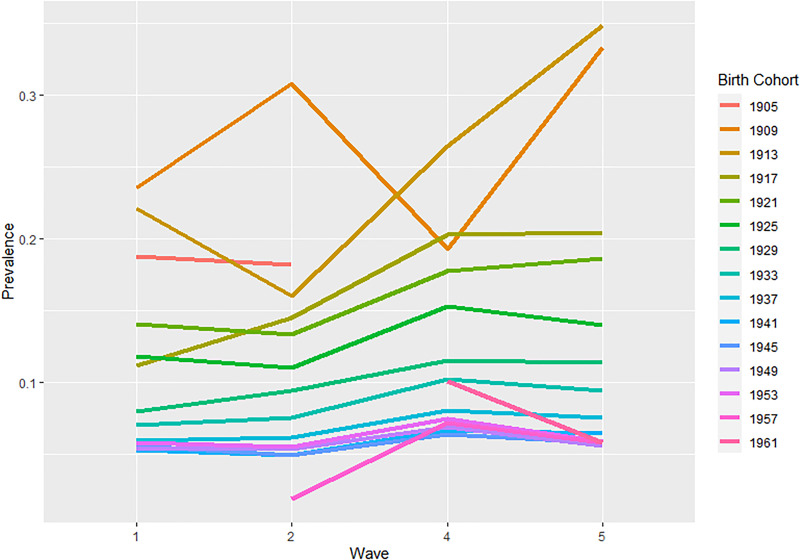
Unadjusted prevalence of suicide ideation by Birth Cohort groups over study time. *Notes:* Wave 1 (2004–2005), Wave 2 (2006–2007), Wave 4 (2011) and Wave 5 (2013). Birth cohort is denoted by the first year of each cohort.

The overall sample consisted of 50 782 participants that were interviewed at least once (*n* = 22 621; 44.5% were men). Descriptive statistics by the presence of suicide ideation are provided in Table 1. A total of 4031 participants (7.94%) showed suicide ideation the first time they were interviewed. Prevalence of suicide ideation across gender, age, level of education, country groups and health status can be consulted in Table 1. Unadjusted prevalence rates of suicide ideation were descriptively higher for cohorts born during the first two decades of the 20th century than the cohorts born since 1941 (Fig. 1). Two cohorts 1909–1912 and 1913–1916, reported descriptively an increasing trend of suicide ideation in the year 2013 (Fig. 1).

**Table 1.** Characteristics of the SHARE participants interviewed in the baseline

	Suicide ideation			
	No (*n* = 46 751)	Yes (*n* = 4031)	*χ^2^/Wilcoxon test*	*p*
Sex, *n* (%)			287.214	<0.001
Men	21 339 (45.6)	1282 (31.8)		
Women	25 412 (54.4)	2749 (68.2)		
Age,			497.89	<0.001
50–64	25 712 (55.0)	1682 (41.7)		
65–79	17 137 (36.7)	1633 (40.5)		
+80	3908 (8.3)	716 (17.8)		
Marital status			834.15	<0.001
Single	2470 (5.3)	232 (5.8)		
Married-cohabiting	34 327 (73.4)	2181 (54.1)		
Separated-divorced	3158 (6.8)	392 (30.4)		
Widow	6792 (14.5)	1226 (30.4)		
Education, *n* (%)			578.855	<0.001
Less than primary	1597 (3.4)	346 (8.6)		
Primary	10 571 (22.8)	1291 (32.3)		
Secondary	25 325 (54.6)	1948 (10.4)		
Tertiary	8926 (19.2)	416 (10.4)		
Health status, Mean (s.d.)	71.82 (15.93)	56.20 (16.70)	2797.205	<0.001
Country			517.231	<0.001
Austria	1477 (3.2)	60 (1.5)		
Belgium	3323 (7.1)	325 (8.1)		
Czech Republic	2501 (5.3)	190 (4.7)		
Denmark	1484 (3.2)	93 (2.3)		
Estonia	5984 (12.8)	531 (13.2)		
France	2525 (5.4)	336 (8.3)		
Germany	2735 (5.9)	163 (4.0)		
Greece	2421 (5.2)	163 (4.0)		
Hungary	2563 (5.5)	384 (9.5)		
Ireland	1049 (2.2)	36 (0.9)		
Israel	2207 (4.7)	226 (5.6)		
Italy	2325 (5.0)	156 (3.9)		
Luxembourg	1429 (3.1)	139 (3.4)		
Netherlands	2658 (5.7)	126 (3.1)		
Poland	2077 (4.4)	295 (7.3)		
Portugal	1770 (3.8)	185 (4.6)		
Slovenia	2491 (5.3)	186 (4.6)		
Spain	2024 (4.3)	263 (6.5)		
Sweden	2823 (6.0)	121 (3.0)		
Switzerland	885 (1.9)	53 (1.3)		

*Notes*: Baseline year varied depending on the country. Baseline assessment in Austria, Belgium, Switzerland, Germany, Denmark, Spain, France, Greece, Italy, Netherlands, Sweden, Israel was in 2004. It was in 2007 in the Czech Republic, Ireland and Poland. It was in 2011 in Estonia, Hungary, Portugal, Slovenia and it was Luxembourg in 2013

**Fig. 2.**
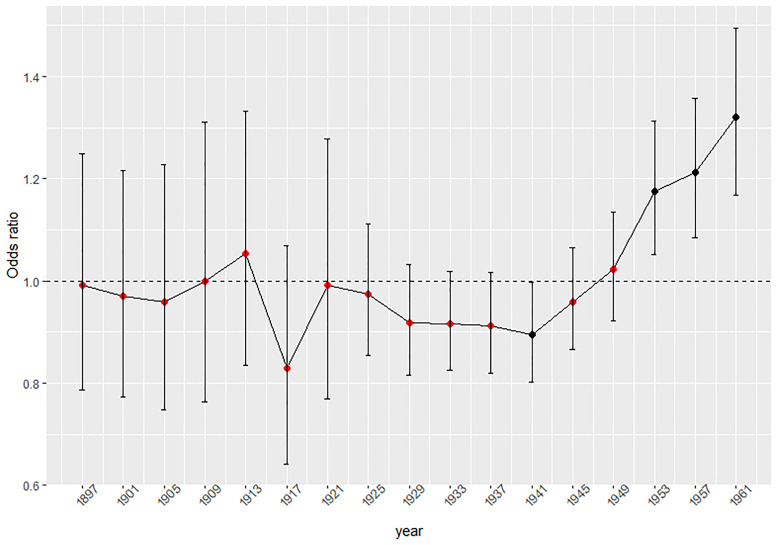
Predicted ORs and 95% CIs of suicide ideation for each birth cohort. *Note*: Births cohorts are denoted by the first year of each cohort.

**Fig. 3.**
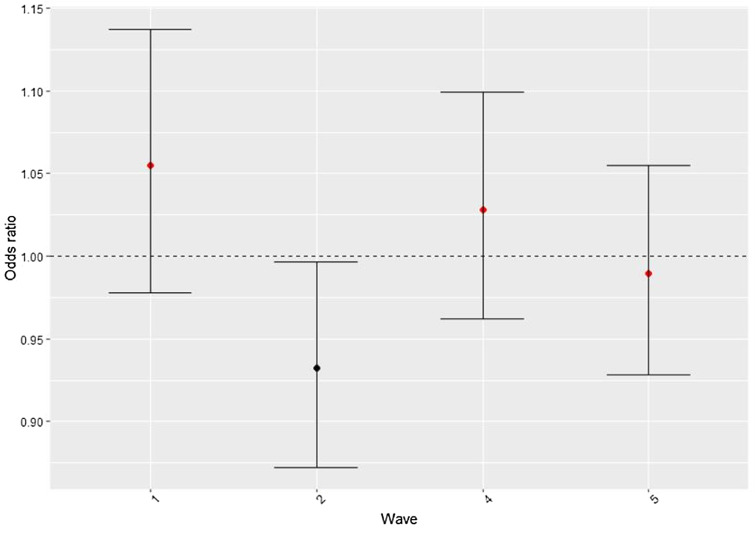
Predicted ORs and 95% CIs of suicide ideation for each time period. *Notes*: Wave 1 covered the 2004–2005 period; Wave 2 was conducted in the 2006–2007 period; Wave 4 was in 2011 and Wave 5 was in 2013.

The results of the mixed-effect regression model are presented in Table 2. As can be seen in the fixed part of the model, youngest-old people (65–79 years) showed a lower likelihood of suicide ideation (*OR* = 0.88; CI 95% = 0.81, 0.96) than middle-aged people (50–64 years) (Table 2). In contrast, people aged 80 + years reported similar levels of suicide ideation than middle-aged people (*OR* = 0.95; CI 95% = 0.91, 1.01). In addition, higher suicide ideation was related to being a woman, having a lower level of education and lower health status. Population with married or cohabiting marital status reported lower suicide ideation than singles (*OR* = 0.72; CI 95% = 0.65, 0.79). Being widowed (*OR* = 1.21; CI 95% = 1.09, 1.35) and separated/divorced (*OR* = 1.27; CI 95% = 1.13, 1.42) were associated with higher odds of suicide ideation than being single. Significant differences in suicide ideation estimates were also found across countries (Table 2). Regarding the random part of the model, most of the variation in suicide ideation was explained by inter-participant characteristics, 57.8% of the total variance (*σ*^2^ = 1.40; CI 95% = 1.24–1.55). Birth-cohort random effect explained a smaller percentage 0.85% but still had an effect on the variability in suicide ideation (*σ*^2^ = 0.02; CI 95% = 0.0019–0.0023), whereas the time period level explained only 0.13% of the variation (*σ*^2^ = 0.003; CI 95% = 0.001–0.006) (Table 2). Predicted estimates for suicide ideation across birth cohort groups are shown in Fig. 2. People born in 1941–1944 (*OR* = 0.89; CI 95% = 0.80–0.99), showed a decreased likelihood of reporting suicide ideation (Fig. 2). In addition, people born between 1953 and 1955 (*OR* = 1.17; CI 95% = 1.05–1.31), 1956–1960 (*OR* = 1.21; CI 95% = 1.08–1.35) and 1961–1964 (OR = 1.32; CI 95% = 1.16–1.49) showed a higher likelihood of reporting suicide ideation. Predicted intervals and values of time period effect are displayed in Fig. 3. In comparison with other time periods, participants assessed during the 2006–2007 period reported a decreased likelihood of suicide ideation (*OR* = 093; CI 95% = 0.87–0.99). The rest of the birth cohorts and period levels did not show any significant effect on suicide ideation.

**Table 2.** Parameter estimates of the multilevel mixed-effects models assessing the prevalence of suicide ideation in the SHARE participants

Mixed model	Suicide ideation	
Fixed effects	OR (CI 95%)	*p*
Intercept	2.108 (1.72, 2.58)	<0.001
Gender (male)	0.82 (0.78, 0.86)	<0.001
Age (ref. 50–64)		
65–79	0.88 (0.81,0.96)	0.004
80+	0.95 (0.91, 1.01)	0.078
Marital status (ref. single)		
Married-cohabiting	0.72 (0.65, 0.79)	<0.001
Separated-divorced	1.27 (1.13,1.42)	<0.001
Widow	1.21 (1.09, 1.35)	<0.001
Education (ref. no education)		
Primary	0.94 (0.85, 1.04)	0.25
Secondary	0.84 (0.76, 0.93)	0.001
Tertiary	0.75 (0.68, 0.85)	<0.001
Health Satus	0.95 (0.9, 0.95)	<0.001
Country (ref. Austria)		
Belgium	2.01 (1.79, 2.26)	<0.001
Czech Republic	1.93 (1.71, 2.17)	<0.001
Denmark	0.89 (0.76, 1.04)	0.15
Estonia	1.13 (0.99, 1.28)	0.057
France	2.77 (2.46, 3.12)	<0.001
Germany	1.32 (1.15, 1.51)	0.001
Greece	0.97 (0.81, 1.16)	0.761
Hungary	2.17 (1.84, 2.55)	<0.001
Ireland	0.73 (0.50, 1.07)	0.105
Israel	1.57 (1.35, 1.84)	<0.001
Italy	0.85 (0.74, 0.97)	0.017
Luxembourg	1.82 (1.45, 2.27)	<0.001
Netherlands	1.13 (0.99, 1.30)	0.097
Poland	1.87 (1.60, 2.19)	<0.001
Portugal	1.47 (1.20, 1.81)	<0.001
Slovenia	1.10 (0.94, 1.29)	0.24
Spain	1.40 (1.23, 1.59)	<0.001
Sweden	0.98 (0.85, 1.14)	0.82
Switzerland	1.49 (1.29, 1.73)	<0.001
Random effects	Variance	Bootstrap 95% CI
Participant	1.40	1.24–1.55
Birth Cohort	0.02	0.0019–0.023
Period	0.003	0.001–0.006

## Discussion

This study is, to our knowledge, the first to disentangle age, cohort and period effects on the prevalence of suicide ideation among people aged 50 years and older in Europe, shedding light on the ongoing debate about the role of ageing in suicide ideation. To do so, we analysed the prevalence trends of suicide ideation, including the age effect and the variability effects of birth cohorts from 1905 to 1961, and of four time periods between 2004 and 2013. In this study, the main effects of suicide ideation prevalence were primarily due to individual-level factors. In comparison with youngest-old (65–79 years), middle-aged (50–64 years) reported higher suicide ideation, regardless of period and cohort effects. Middle-aged might be a period associated with economic, health and social burdens that could rise the prevalence of suicidal ideation (Jo *et al*., 2017). In contrast, no differences were found between people aged 80 years and older and those in middle-aged. Oldest-old people might more likely have to face with difficulties such as physical diseases and bereavement than youngest-old people (Koo *et al*., 2017), which may increase their likelihood of suicide ideation. Our results support that the relationship between age and suicide ideation may not be linear in elderly people, and suggest that different age groups should be separately analysed in the study of suicide ideation in older people.

In line with previous literature, married reported a lower likelihood of suicide ideation than single people (Smith *et al*., 1988). In contrast, being single was associated with a lower level of suicidal ideation than being widowed and separated-divorced.

Our study has also revealed that there was a small but still relevant cohort effect in the prevalence of suicide ideation. Decreasing trends of suicide ideation were observed since the earliest 20s, with cohorts born between 1941 and 1944 reporting the lowest likelihood of suicide ideation. This result is congruent with other studies that have shown that people born just before and during World War II were the generation with the lowest prevalence of suicide deaths and depression (Phillips, 2014; Sullivan *et al*., 2020). Some authors argue that people from this generation could have learned to adapt personal aspirations to persevere through difficult times and be more resilient to frustrations (Phillips, 2014). Other authors have also supported that selective mortality could have largely affected this generation, i.e. those with health problems died younger and hence they are not presented in middle and old aged population studies (Hasin and Link, 1988). Further studies should explore the reasons why so many people from this generation have shown low levels of suicide ideation.

On the other hand, an increasing trend of suicide ideation was observed for post-War generations (called baby boomers), reaching the significant level for people born from 1953 to 1964. This finding is also congruent with previous studies that report that baby boomers are showing higher psychopathology than their ancestors (Phillips, 2014). According to Durkheim (2005) societal and cultural changes that occurred from the post-war generations onwards might have weakened societal bonds, making these generational groups more vulnerable to suicide. Other authors have added that baby boomers were the first ones who imposed a youth culture where ageing was marginalised. Therefore, it is possible that some of them became victims of the culture they created themselves (McCue and Balasubramaniam, 2016). Although further studies are necessary, our findings suggest that baby-boomers are more affected by suicide ideation. Therefore, there will probably be a higher need for elderly mental health and social community support in Europe in the future since this generation is already entering the old age.

Finally, our study also showed that a very small amount of suicide ideation changes might be attributed to period effects. A small period effect was observed for participants assessed during the 2006–2007 years, who reported a lower prevalence of suicide ideation in comparison with other time occasions. Some authors have highlighted the optimism experienced during the 2006–2007 years in the European Union due to the inclusion of new countries and to good economic stability which met or even exceeded expectations (Schrogl *et al*., 2008). Conversely, no increases of suicide ideation were observed during the economic downturn period in the 2011 and 2013 years. Although we do not have a clear interpretation for this result, it is possible that our cross-country approach blurred some differences, since some European countries were greatly affected by the economic recession, whereas others were not. In addition, the effect of a selection bias cannot be ruled out either, since people may be less likely to participate in a study if they were affected by the events.

The great variety of countries covered, the use of a longitudinal panel design – which allowed us to simultaneously manage age, period and cohort effects – controlling for important effects such as general health or country and the use of a measure that recorded the presence of suicide ideation in the last month – which reduced the likelihood of recall bias effects – are probably the main strengths of this study. However, we acknowledge that the results of this study should also be interpreted with caution due to the following limitations. Firstly, although European countries share common cultural and societal values, it is possible that the aim of searching a global European birth and period cohort effect may have hidden the variability that each birth cohort and period group had in the different countries. Unfortunately, our data did not allow specific country analyses due to the variability of time periods that each country included in the study. Secondly, our study did not include information of severity and covered a concept of passive suicide ideation, which has sometimes been considered as a less severe form of suicide ideation in clinical settings. However, wishes of death or that life is not worth living have been strongly associated with psychopathology and they did not seem to be normative in older populations (Van Orden *et al*., 2015). Thirdly, the use of a self-reported measure might have introduced biases related to underreporting wishes of death due to social desirability. Fourthly, household response rates were globally higher than 45% in all the study waves. However, there were some countries with rates lower than 40%. This fact has to be carefully considered in the interpretation of the study results. Nonetheless, it is important also to consider that response rates in the SHARE study are in line with the numbers of comparable European surveys conducted in the same period (Koen *et al*., 2018). Fifthly, the method on how to best deal with point identification problem is still an open debate in the literature with not a single answer (O'Brien, 2017). We acknowledge there are other approaches based on mathematical solutions that could have been applied (Tu *et al*., 2012). However, we managed the point identification problem according to explicit theory-based assumptions that have also been shown as valid (Reither *et al*., 2015). Moreover, we categorised age, periods and cohorts into unequal width groups in order to also decrease the possible linear dependence between age, period and cohort effects. Finally, although the study included a follow-up period of 8 years (2004–2013), middle ages could be underrepresented for earlier generations.

In spite of all these limitations, our study is the first to analyse the role of ageing including the age, cohort and period effects on the prevalence of suicide ideation in the European population. The findings in our study support that the relationship between age and suicide ideation is non-linear. Middle-age may be a particularly high-risk time period of suicide ideation in comparison with early elderly (65–78 years). However, the protective effect of older age might not apply for people age 80+. Our results have also suggested that there is a certain cohort effect, with baby boomers being more likely to report a wish to die than the generation born just during War World II, showing this later generation the lowest levels of suicide ideation. Thus, it is possible that a greater need for community mental health services by older European people could follow in the near future.

## Supplementary material

For supplementary material accompanying this paper visit http://dx.doi.org/10.1017/S2045796021000020. click here to view supplementary material

## Availability of Data and Materials

This paper uses data from SHARE Waves 1, 2, 4, and 5 (DOIs:10.6103/SHARE.w1.700, 10.6103/SHARE.w2.700, 10.6103/SHARE.w4.700, and 10.6103/SHARE.w5.700), which are available via the SHARE project's website.

## References

[ref1] Almeida OP, Draper B, Snowdon J, Lautenschlager NT, Pirkis J, Byrne G, Sim M, Stocks N, Flicker L and Pfaff JJ (2012) Factors associated with suicidal thoughts in a large community study of older adults. The British Journal of Psychiatry 201, 466–472. 23209090 10.1192/bjp.bp.112.110130

[ref2] Bates D, Mächler M, Bolker B and Walker S (2015) Fitting linear mixed-effects models using lme4. Journal of Statistical Software. Articles 67, 1–48.

[ref3] Bergmann M, Kneip T, De Luca G and Scherpenzeel A (2017) Survey participation in the survey of health, ageing and retirement in Europe (SHARE), Wave 1–6. Based on Release 6.0.0 (March 2017), SHARE Working Paper Series 31-2017. Munich: Munich Center for the Economics of Aging, pp. 1–46.

[ref4] Bernal M, Haro JM, Bernert S, Brugha T, de Graaf R, Bruffaerts R, Lépine JP, de Girolamo G, Vilagut G and Gasquet I (2007) Risk factors for suicidality in Europe: results from the ESEMED study. Journal of Affective Disorders 101, 27–34. 17074395 10.1016/j.jad.2006.09.018

[ref5] Borges G, Angst J, Nock MK, Ruscio AM and Kessler RC (2008) Risk factors for the incidence and persistence of suicide-related outcomes: a 10-year follow-up study using the National Comorbidity Surveys. Journal of Affective Disorders 105, 25–33. 17507099 10.1016/j.jad.2007.01.036 PMC2248274

[ref6] Börsch-Supan A, Hank K and Jürges H (2005) A new comprehensive and international view on ageing: introducing the “survey of health, ageing and retirement in Europe”™. European Journal of Ageing 2, 245–253. 28794739 10.1007/s10433-005-0014-9 PMC5546288

[ref7] Börsch-Supan A, Brugiavini A, Jürges H, Kapteyn A, Mackenbach J, Siegrist J and Weber G (2008) First results from the Survey of Health, Ageing and Retirement in Europe (2004–2007): Starting the longitudinal dimension. Mannheim, Germany: Mannheim Research Institute for the Economics of Aging.

[ref8] Börsch-Supan A, Brandt M, Hunkler C, Kneip T, Korbmacher J, Malter F, Schaan B, Stuck S and Zuber S (2013) Data resource profile: the survey of health, ageing and retirement in Europe (SHARE). International Journal of Epidemiology 42, 992–1001. 23778574 10.1093/ije/dyt088 PMC3780997

[ref9] Briggs R, Tobin K, Kenny RA and Kennelly SP (2018) What is the prevalence of untreated depression and death ideation in older people? Data from the Irish Longitudinal Study on aging. International Psychogeriatrics 30, 1393–1401. 29335038 10.1017/S104161021700299X

[ref10] Caballero FF, Soulis G, Engchuan W, Sanchez-Niubo A, Arndt H, Ayuso-Mateos JL, Haro JM, Chatterji S and Panagiotakos DB (2017) Advanced analytical methodologies for measuring healthy ageing and its determinants, using factor analysis and machine learning techniques: the ATHLOS project. Scientific Reports 7, 43955. 28281663 10.1038/srep43955 PMC5345043

[ref11] Cabello M, Miret M, Ayuso-Mateos J, Caballero FF, Chatterji S, Tobiasz-Adamczyk B, Haro JM, Koskinen S, Leonardi M and Borges G (2019) Cross-national prevalence and factors associated with suicide ideation and attempts in older and young-and-middle age people. Aging & Mental Health 24, 1533–1542. 30990056 10.1080/13607863.2019.1603284

[ref12] de la Fuente J, Caballero FF, Sánchez-Niubó A, Panagiotakos DB, Prina AM, Arndt H, Haro JM, Chatterji S and Ayuso-Mateos JL (2018) Determinants of health trajectories in England and the United States: an approach to identify different patterns of healthy aging. The Journals of Gerontology: Series A 73, 1512–1518. 10.1093/gerona/gly006 PMC6175023 29346518

[ref13] Durkheim E (2005) Suicide: A Study in Sociology. London: Taylor & Francis.

[ref14] Eurostat (2020) Suicide death rate by age group. Available at https://ec.europa.eu/eurostat/web/products-datasets/-/tps00202 (Accessed 12 April 2020).

[ref15] Fässberg MM, Vanaelst B, Jonson M, Sterner TR, Ahlner F, Wetterberg H, Rydén L, Kern S, Sigström R and Zettergren A (2020) Epidemiology of suicidal feelings in an ageing Swedish population: from old to very old age in the Gothenburg H70 birth cohort studies. Epidemiology and Psychiatric Sciences 29, e26, 1–14. 10.1017/S2045796019000143 PMC8061288 30929647

[ref16] Hasin D and Link B (1988) Age and recognition of depression: implications for a cohort effect in major depression. Psychological Medicine 18, 683–688. 3263662 10.1017/s0033291700008369

[ref17] Hubers AA, Moaddine S, Peersmann SH, Stijnen T, Van Duijn E, Van der Mast RC, Dekkers OM and Giltay EJ (2016) Suicidal ideation and subsequent completed suicide in both psychiatric and non-psychiatric populations: a meta-analysis. Epidemiology and Psychiatric Sciences 27, 186–198. 27989254 10.1017/S2045796016001049 PMC6998965

[ref18] Jo A, Jeon M and Oh H (2017) Age-differentiated risk factors of suicidal ideation among young and middle-aged Korean adults. Osong Public Health and Research Perspectives 8, 201. 28781943 10.24171/j.phrp.2017.8.3.07 PMC5525562

[ref19] Knowles JE and Frederick C (2016) Mertools: tools for analyzing mixed effect regression models. R Package Version *0.3.0*. https://CRAN.R-project.org/package=merTools (Accessed 19 May 2020).

[ref20] Koen B, Loosveldt G, Vandenplas C and Stoop I (2018) Response rates in the European Social Survey: increasing, decreasing, or a matter of fieldwork efforts? Survey Methods: Insights from the Field, 1–12. https://surveyinsights.org/?p=9673 (Accessed 11 April 2020).

[ref21] Koo YW, Kõlves K and De Leo D (2017) Suicide in older adults: differences between the young-old, middle-old, and oldest old. International Psychogeriatrics 29, 1297. 28511737 10.1017/S1041610217000618

[ref22] Malter F and Börsch-Supan A (2013) SHARE Wave 4: Innovations & methodology. Munich: Munich Center for the Economics of Aging, Max Planck Institute for Social Law and Social Policy.

[ref23] Malter F and Börsch-Supan A (2015) SHARE Wave 5: Innovations & methodology. Munich: Munich Center for the Economics of Aging, Max Planck Institute for Social Law and Social Policy.

[ref24] McCue RE and Balasubramaniam M (eds) (2016) Rational Suicide in the Elderly: Clinical, Ethical, and Sociocultural Aspects. Cham, Switzerland: Springer.

[ref25] O'Brien RM (2017) Mixed models, linear dependency, and identification in age-period-cohort models. Statistics in Medicine 36, 2590–2600. 28378504 10.1002/sim.7305

[ref26] O'Riley AA, Van Orden K and Conwell Y (2014) Suicidal ideation in late life. In Pachana NA and Laidlaw K (eds), The Oxford Handbook of Clinical Geropsychology: International Perspectives. Oxford, UK: Oxford University Press, pp. 267–284.

[ref27] Phillips JA (2014) A changing epidemiology of suicide? The influence of birth cohorts on suicide rates in the United States. Social Science & Medicine 114, 151–160. 24929916 10.1016/j.socscimed.2014.05.038

[ref28] Prince MJ, Reischies F, Beekman AT, Fuhrer R, Jonker C, Kivela S, Lawlor BA, Lobo A, Magnusson H and Fichter M (1999) Development of the EURO–D scale–a European Union initiative to compare symptoms of depression in 14 European centres. The British Journal of Psychiatry 174, 330–338. 10533552 10.1192/bjp.174.4.330

[ref29] R Core Team (2013) R: A Language and Environment for Statistical computing. Vienna: R Core Team. http://www.R-project.org/.

[ref30] Reither EN, Masters RK, Yang YC, Powers DA, Zheng H and Land KC (2015) Should age-period-cohort studies return to the methodologies of the 1970s? Social Science & Medicine 128, 356–365. 25617033 10.1016/j.socscimed.2015.01.011 PMC4357521

[ref31] Schrogl K, Mathieu C and Peter N (2008) Yearbook on Space Policy 2006/2007: New Impetus for Europe. Vienna: Springer Science & Business Media.

[ref32] Smith JC, Mercy JA and Conn JM (1988) Marital status and the risk of suicide. American Journal of Public Health 78, 78–80. 3337311 10.2105/ajph.78.1.78 PMC1349216

[ref33] Stolz E, Fux B, Mayerl H, Rásky É and Freidl W (2016) Passive suicide ideation among older adults in Europe: a multilevel regression analysis of individual and societal determinants in 12 countries (SHARE). Journals of Gerontology Series B: Psychological Sciences and Social Sciences 71, 947–958. 10.1093/geronb/gbw041 PMC4982389 27048569

[ref34] Sullivan KJ, Liu A, Dodge HH, Andreescu C, Chang CH and Ganguli M (2020) Depression symptoms declining Among older adults: birth cohort analyses from the rust belt. The American Journal of Geriatric Psychiatry 28, 99–107. 31300193 10.1016/j.jagp.2019.06.002 PMC6898763

[ref35] Tu Y, Krämer N and Leec W (2012) Addressing the identification problem in age-period-cohort analysis: a tutorial on the use of partial least squares and principal components analysis. Epidemiology (Cambridge, Mass.) 23, 583–593. 10.1097/EDE.0b013e31824d57a9 22407139

[ref36] Twenge JM, Cooper AB, Joiner TE, Duffy ME and Binau SG (2019) Age, period, and cohort trends in mood disorder indicators and suicide-related outcomes in a nationally representative dataset, 2005–2017. Journal of Abnormal Psychology 128, 185–199. 30869927 10.1037/abn0000410

[ref37] Van Orden KA, O´Riley AA, Simning A, Podgorski C, Richardson TM and Conwell Y (2015) Passive suicide ideation: an indicator of risk among older adults seeking aging services? The Gerontologist 55, 972–980. 24714844 10.1093/geront/gnu026 PMC4668765

[ref38] Wickham H (2016) ggplot2: Elegant Graphics for Data Analysis. New York, NY: Springer.

