# Supplementary material for: The role of ageing in the wish to be dead: disentangling age, period and cohort effects in suicide ideation in European population
Source: Epidemiol Psychiatr Sci. 2021 Feb 18;30:e17. doi: 10.1017/S2045796021000020 (PMC8061246; doi:10.1017/S2045796021000020)
Supplement: Supplementary file 1 [file S2045796021000020sup001.docx]

Table S1. Household reponse rates by Wave and country

|  | Wave 1 | Wave 2 | Wave 4 | Wave 5 |
| --- | --- | --- | --- | --- |
| *Austria* | 51.9% | -- | 97% | -- |
| *Belgium* | 40.3% | 42.1% | 42.7% | 36.6% |
| *Czech Republic* | -- | 72% | 57.4% | 62.0% |
| *Denmark* | 67.1% | 65.2% | 51.6% | 59.6% |
| *Estonia* | -- | -- | 62.8% | -- |
| *France* | 97.5% | 95.3%% | 64.8% | -- |
| *Hungary* | -- | -- | 60.6% | -- |
| *Germany* | 58.2% | 48.8% | -- | 34.9% |
| *Greece* | 68.7% | 58.2% | -- | -- |
| *Israel* | 64.5% | 78.6% | -- | 67.5% |
| *Italy* | 55.2% | 50.9% | 40.9% | 43.4% |
| *Luxembourg* | -- | -- | -- | 32.7% |
| *Netherlands* | 61.3% | 47.5% | 41.1% | 49.7% |
| *Poland* | -- | 55.2% | -- | -- |
| *Portugal* | -- | -- | 61.9% | -- |
| *Slovenia* | -- | -- | -- | 45.8% |
| *Spain* | 51.1% | 58.7% | 63.3% | 61.9% |
| *Sweden* | 53.9% | 35.7% | -- | 39.4% |
| *Switzerland* | 44.0% | 65.8% | 54.9% | -- |
| *Total* | 60.1% | 61.6% | 62.2% | 46.2% |

Table S2: Number of observations by period and countries

|  | Periods (years) | | | |
| --- | --- | --- | --- | --- |
| Countries | 2004-2005 | 2006-2007 | 2011 | 2013 |
| *Austria* | 1547 | 1172 | 5182 | 4337 |
| *Belgium* | 3671 | 3067 | 5241 | 5659 |
| *Czech Republic* | 0 | 2734 | 6031 | 5826 |
| *Denmark* | 1597 | 2520 | 2332 | 4177 |
| *Estonia* | 0 | 0 | 6690 | 6000 |
| *France* | 3032 | 2843 | 5737 | 4507 |
| *Germany* | 2930 | 2523 | 1612 | 5550 |
| *Greece* | 2654 | 3053 | 0 | 0 |
| *Hungary* | 0 | 0 | 2996 | 0 |
| *Ireland* | 0 | 1107 | 0 | 0 |
| *Israel* | 2487 | 2429 | 0 | 2465 |
| Italy | 2493 | 2911 | 3609 | 4764 |
| *Luxembourg* | 0 | 0 | 0 | 1590 |
| *Netherlands* | 2842 | 2607 | 2781 | 4161 |
| *Poland* | 0 | 2414 | 1868 | 0 |
| *Portugal* | 0 | 0 | 2010 | 0 |
| *Slovenia* | 0 | 0 | 2710 | 2955 |
| *Spain* | 2343 | 2176 | 3610 | 6560 |
| *Sweden* | 2984 | 2702 | 2116 | 4669 |
| *Switzerland* | 945 | 1418 | 3671 | 3011 |
